# Unraveling the Role of Foods on Chronic Anti- and Pro-Inflammatory Cytokines: A Systematic Review of Chronic Dietary Intervention Trials in Humans

**DOI:** 10.3390/nu17172834

**Published:** 2025-08-30

**Authors:** Veronica D’Antonio, Marina Ramal-Sanchez, Chiara Bravo-Trippetta, Elena Corvaglia, Mauro Serafini, Donato Angelino

**Affiliations:** Department of Bioscience and Technology for Food, Agriculture and Environment, University of Teramo, 64100 Teramo, Italy; vdantonio@unite.it (V.D.); mramalsanchez@unite.it (M.R.-S.); cgbravotrippetta@unite.it (C.B.-T.); ecorvaglia@unite.it (E.C.); dangelino@unite.it (D.A.)

**Keywords:** diet, food, cytokines, inflammation, food intervention, chronic disease, non-communicable disease, low-grade inflammation, anti-inflammatory, pro-inflammatory

## Abstract

Background/Objectives: Unbalanced diets contribute to a rise in low-grade systemic inflammation, a risk factor for metabolic diseases. The aim of this study is to systematically review evidence from chronic intervention studies to understand the role of foods in modulating inflammatory responses in humans. Methods: A literature search was conducted on PubMed using specific keywords. Risk of bias was assessed using Cochrane guidelines. Inclusion criteria required chronic dietary intervention studies measuring cytokine levels in humans. Results: In the 75 studies selected, results revealed extremely high variability both in outcomes, study design, and participant selection criteria. Studies with fruits and vegetables showed a reduction in circulating cytokine levels and/or an increase in anti-inflammatory cytokines in 80% of studies (8/10), followed by fish (78%; 7/9), dairy (67%; 4/6), cereals (64%; 7/11), and oils (57%; 4/7). Beverages and hot beverages showed a decrease in circulating cytokines in 50% of cases (10/20 and 4/8, respectively). An increase in pro-inflammatory cytokines was observed in dietary interventions with beverages. As further findings, we also observed greater effectiveness from fruits and vegetables (87.5%; 7/8), fish (75%; 6/8), and cereals (62.5%; 5/8) when studies were conducted in subjects with pathologies or risk factors. Conclusions: Fruits and vegetables, fish, and cereals reduce systemic inflammation mainly in subjects with pathologies or risk factors. However, the limited number of studies do not allow us to draw solid conclusions on individual foods. Standardized dietary intervention trials are urgently needed to understand the role of foods in modulating inflammatory responses and to deliver findings to the general public.

## 1. Introduction

Over recent decades, unhealthy lifestyle factors have driven a rise in chronic metabolic diseases like obesity, diabetes, and cardiovascular diseases (CVDs) [1,2]. An analysis of the worldwide effects of unbalanced dietary habits—such as excessive sodium consumption, inadequate whole grain intake, and insufficient fruit and vegetable consumption—indicates that they contributed to 11 million deaths and 255 million disability-adjusted life years [2].

A common factor in non-communicable diseases (NCDs) is low-grade systemic inflammation, which disrupts immune function and promotes the onset and/or progression of chronic pathologies such as metabolic syndrome, type 2 diabetes (T2D), and CVD through different mechanisms [3]. For instance, inflammation encourages the development of atherosclerotic plaques and their instability, which can lead to myocardial infarction [4,5]. Similarly, pro-inflammatory factors can also contribute to tissue-specific insulin resistance through the disruption of insulin signaling pathways, promoting the development of diabetes [6]. Given the critical role of inflammation in NCDs, understanding how diet influences inflammatory processes is essential. Adults who adhere to diets rich in foods that are considered as pro-inflammatory are more likely to develop certain cancers, cardiovascular disease, disorders related to mental health, and musculoskeletal diseases [7]. On the contrary, interventions with healthy dietary patterns, such as the Mediterranean diet, are rich in fruits, vegetables, and omega-3 fatty acids and have been linked to a decrease in systemic inflammation markers and a drop in cardiovascular event rates in high-risk participants [8], as well as lower inflammation levels in cross-sectional observational studies [9]. In the context of inflammation, it is critical to assess the effects of diet and specific foods on cytokines, which are low-molecular-weight proteins that play important roles in controlling inflammatory processes by modulating immune responses. Their specific functions differ depending on the source of secretion, target cells, and phase of the immune response in which they are active [10]. These molecules, which include tumor necrosis factors (TNFs), interleukins (ILs), interferons (IFNs), and others, can be classified as pro-inflammatory (e.g., TNF-α, IL-8) or anti-inflammatory (e.g., IL-10, tumor growth factor beta, TGF-β) [11,12]. Additionally, some cytokines, such as IL-6, can show either pro-inflammatory or anti-inflammatory function depending on the types of immune cells involved [13]. Imbalances between pro-inflammatory and anti-inflammatory cytokines are recognized as a link between diet and health [14]. In fact, inflammation related to diet can manifest in different ways, one of which is postprandial inflammation, a short-term, regulated immune response to food intake that occurs particularly after food ingestion. It typically resolves within a few hours, but it is particularly marked after a meal with elevated fat content (high fat meal, HFM), which has been observed to raise postprandial levels of IL-6 within 4–8 h of consumption [15]. While these meals elevate inflammatory markers, incorporating vegetable products—such as fruit juice [16]—can contribute to mitigating these effects. Additionally, plant-based energy- and macronutrient-matched meals ameliorate postprandial stress compared to conventional HFMs in T2D and obese patients [17]. Repeated dietary acute stressors significantly increase risk factors for CVD. When the inflammatory response becomes exaggerated or prolonged, it can lead to chronic low-grade inflammation and thus health issues. Frequent red meat consumption has been associated with an inflammatory pattern, characterized by an increase in IL-6 and IL-8; IL-8 levels were also increased with the frequent intake of sweets, while a higher intake of shelled fruits correlated with lower levels of IL-6 [18]. While this evidence offers valuable insights, many of the available studies are epidemiological ones or overly short-term intervention trials, making it difficult to establish causality between the consumption of specific foods and inflammation. To date, no comprehensive synthesis of intervention studies has been conducted to clarify the effects of various foods and food categories on circulating cytokines in humans. The aim of this study is to systematically review evidence from chronic intervention studies in humans to understand the role of foods in modulating inflammatory responses, as mediated by circulating cytokines.

## 2. Materials and Methods

### 2.1. Search Strategy and Study Selection

The primary objective of this study was to identify studies that investigated whether chronic dietary interventions involving whole or processed foods—excluding supplements and extracts, or complex dietary patterns—modulate inflammation, specifically cytokine levels, in human subjects.

The search strategy of the study was carried out as follows: at first, a systematic search was carried out using the PubMed database (National Library of Medicine, Bethesda, MD, USA; https://pubmed.ncbi.nlm.nih.gov) and the following keywords: (cytokines [MeSH]) AND (diet, food and nutrition [MeSH]).

The search was carried out in September 2024, with no limit ranges for the year of publication and with the “Clinical trials” and “Humans” filters activated. Articles were screened and analyzed to select studies in English that met our inclusion criteria: chronic intervention studies, measuring cytokine levels in human participants and intervention studies evaluating the effects of food consumption.

Non-relevant studies were identified based on the following criteria: non-English language, studies not conducted on humans, and short-term studies, as well as studies solely assessing the nutritional status of a selected population, non-relevant interventions or non-eligible participants. Studies involving heavy modifications of the habitual diets of participants were excluded as well. Non-relevant interventions included studies not directly focused on food or nutrition, for example, those focused on drugs, supplements, extracts, alcoholic beverages, fasting, breast milk and physical exercise, or involving enteral or parenteral nutrition or studies on dietary patterns. Participants with severe pathologies (e.g., cancer, multiple sclerosis, kidney failure) or food allergies, pregnant or lactating women, non-adults, and athletes were indicated as non-eligible.

### 2.2. Risk of Bias

The assessment of risk of bias in both individual studies and across all the studies was conducted independently by two authors (V.D.A. and C.B.T.), adhering to the guidelines outlined in the Cochrane Handbook for Systematic Reviews of Interventions, version 5.1.0 [19]. The evaluation of the information concerned the following aspects of bias: selection bias (including sequence generation and allocation concealment); performance bias (blinding of participants and study personnel), detection bias (blinding of outcome assessment), attrition bias (handling of incomplete outcome data), reporting bias (selective reporting of results), and other potential sources of bias. Each study was categorized as having (i) low risk, (ii) unclear risk, or (iii) some or high risk of bias, depending on the adequacy of reported details, for each of the considered criteria. In cases of disagreement, a third author (D.A.) facilitated consensus.

## 3. Results

**Figure 1:**
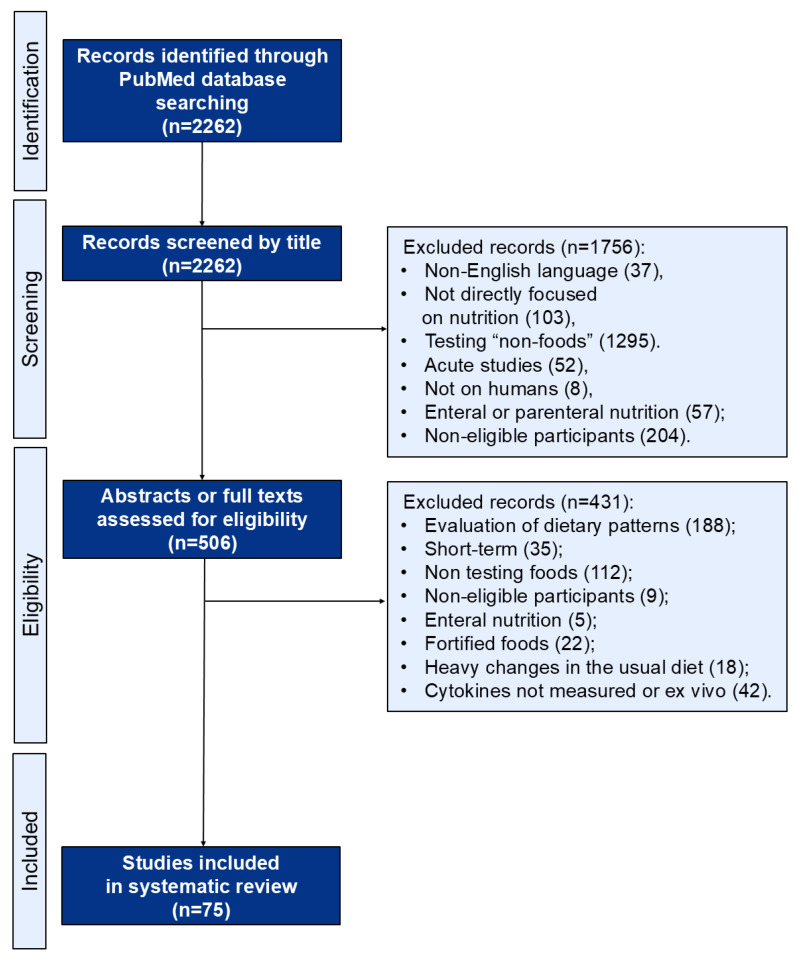
Flow diagram of the study selection process.

Figure 1 shows the flow diagram of the study selection process. The search yielded 2262 results, and the screening of paper titles resulted in the exclusion of 1756 studies for being not in the English language (37) or not conducted on humans (8), or for featuring non-relevant interventions (1507) or non-eligible participants (204). Subsequently, a comprehensive screening of abstracts and full texts was performed on the remaining 506 studies. This step resulted in the exclusion of 431 studies that involved non-eligible participants (9), non-relevant interventions (362), further modifications in the usual diet (18), or a lack of assessment of circulating cytokines (42).

The selection process led to the inclusion of 75 articles. The studies were divided into two main groups: one group of studies focused on the ingestion of solid foods (a single food or various from the same category) and another one on beverages. The different study arms were considered as treatments or controls based on how they were reported by the study itself. Among the selected studies, 49 focused on the chronic ingestion of solid foods (Table 1, Table 2, Table 3, Table 4, Table 5, Table 6 and Table 7) and 26 on beverages (Table 8 and Table 9). Among these, three studies analyzed the effects of two different food categories; therefore, they were separated and discussed in their respective sections according to the food groups involved in each intervention arm.

The most extensively investigated food groups were fruits and vegetables, with a total of 28 interventions: 9 focusing on foods and 19 on beverages. Cereals were tested in 10 interventions, followed by oils and fats, seeds and nuts, hot beverages, and fish, all with 7 studies each, and then dairy and miscellaneous foods in 6 interventions.

Regarding the food items, the majority of the articles have focused on specific single foods, while only 12 studies explored various foods from the same category, such as cereal-based products. Whole grains were the most commonly mentioned food category (seven studies), followed by fruits and vegetables (three studies), dairy (two studies) and red meat in one study. Among specific items, flaxseed and cherries were the most frequently studied, with five papers each, examined as whole seeds (four studies) or oil (one study) for flaxseeds and as a whole food (one study) or beverages (four studies) for cherries. Olive oil and salmon follow with four studies each, while cranberries were examined in three studies. Other foods that were cited more than once included tomatoes, bilberries, coffee, tea, and apple juice, each mentioned in two studies.

### 3.1. Foods

#### 3.1.1. Cereals

Eight of the ten included studies [20,21,22,23,24,25,26,27] compared whole grains to refined grains, one compared ancient versus modern wheat [20], and another examined Kamut [21] (Table 1). Altogether, the studies enrolled 335 participants. Overall, cytokines decreased in six out of ten studies. Conversely, in four studies comparing whole and refined grains, no significant changes in cytokine levels were detected in different individual cohorts: adults with low whole grain intake [22], post-menopausal women [23], overweight/obese individuals with metabolic syndrome [24], and overweight/obese individuals [25].

**Table 1:** **Summary of randomized controlled trials investigating the effects of cereals on circulating cytokines**. Each study includes details of the design, duration, treatment, dose, study population, and reported outcomes. Results are presented as changes within groups and between groups.

Study Design (Duration)	Treatment	Serving Size	Study Population	Population Number (F)	Results Within Groups	Results Between Groups	Reference
Randomized, controlled, crossover, (6 wk × 2)	T: whole grain products C: refined grain products	T: whole grain products > 80 g/d C: whole grain products < 16 g/d	Normal weight, overweight, obese,	33 (31)	T: IL-1β, IL-6, IL-8, IL-10, TNF-α ↔ C: IL-1β, IL-6, IL-8, IL-10, TNF-α ↔	(n.d.)	[22]
Randomized, controlled, crossover, open label (6 wk × 2)	T: whole grains C: refined cereal	112 g/d	Overweight/obese + risk factors (↑ plasma insulin levels, ↑ fasting glucose concentration, ↑ serum triglycerides, ↓ HDL cholesterol, or borderline hypertension)	30 (22)	T: IL-6 ↔ C: IL-6 ↔	T vs. C: IL-6 ↔	[25]
Randomized, crossover, double-blind (4 wk × 2)	T: whole grain products C: refined grain products	30 g/d breakfast cereals, puffed rice/barley, rusks or biscuits 80 g/d pasta	Post menopausal women; healthy.	13 (13)	T: IL-6, IL-1β, TNF-α ↔ C: IL-6, IL-1β, TNF-α ↔	T vs. C: IL-6, IL-1β, TNF-α ↔	[23]
Randomized, parallel, controlled (6 wk)	T: whole grains C: refined grains	3 servings/d	Overweight, obese Low intakes of fruit, vegetables, and whole grains	31 (18)	T1: TNF-α ↓; IL-6 ↔ C: TNF-α ↔; IL-6 ↔	(n.d.)	[26]
Randomized, crossover (4 wk × 3)	T1: barley (18.7 g total dietary fiber) T2: barley + brown rice (11.5 g total dietary fiber) T3: brown rice (4.4 g total dietary fiber)	T1: 60 g T2: 30 + 30 g T3: 60 g	Healthy	28 (17)	T1: IL-6 ↔ (women ↓) T2: IL-6 ↓ (women ↓) T3: IL-6 ↔ (women ↓)	(n.d.)	[27]
Randomized, controlled, crossover, single-blind (8 wk × 2)	T: whole grain products C: refined grain products	Whole grain products: T: >75 g/d C: <10 g/d	Overweight/obese; at risk of metabolic syndrome	50 (32)	(n.d.)	T vs. C: IL-6, IL1β ↓ TNF-α ↔	[28]
Randomized, controlled, crossover, double-blind (6 wk × 2)	T: ancient wheat products C: modern wheat products	500 g/wk of pasta, 150 g/d of bread , 500 g/month of crackers, 1 kg/month of biscuits	Moderate IBS	20 (13)	T: IL-4, IL-6, IL-17, IFN-γ, MCP-1, ↓ IL-8, IL-10, IL-12, IP-10, MIP-1β, TNF-α ↔ C: IL-4, IL-6, IL-8, IL-10, IL-12, IL-17, IFN-γ, IP-10, MIP-1β, MCP-1, TNF-α ↔	(n.d.)	[20]
Randomized, controlled, crossover, single-blind (8 wk × 2)	T: Kamut C: semi-whole-grain wheat	500 g/wk of pasta 150 g/d of bread 500 g/month of crackers 1 kg/month of biscuits	Normal weight/overweight; healthy	22 (14)	T: IL-6, IL-12, MCP-1, MIP-1β, TNF-α ↓ IL-1ra, IL-4, IL-8, IL-10, IL-17, IP-10 ↔ C: MCP-1, MIP-1β ↓ IL-1ra, IL-4, IL-6, IL-8, IL-10, IL-12, IL-17, IP-10, TNF-α ↔	(n.d.)	[21]
Randomized, controlled, parallel (12 wk)	T: whole-grain products C: refined products	60–80% of the daily carbohydrate intake from cereal products	Overweight/obese; metabolic syndrome	40 (23)	(n.d.)	T vs. C: TNF-α, IL-6, IL-1ra ↔	[24]
Randomized, controlled, parallel, single blind (investigators) (8 wk)	T: whole-grain wheat products C: refined wheat products	T: 70 g/d (3 biscuits/d) of whole grain wheat C: 33 g of crackers and 27 g toasted bread	Overweight/obese; healthy; low intake of wholegrain, fruits and vegetables and sedentary lifestyle	68 (45)	T: TNF-α (8 wk) ↓, IL-10 (4 wk) ↑, IL-6 ↔ C: TNF-α, IL-10, IL-6 ↔	T vs. C: TNF-α (8 wk) ↓, IL-10 (4 wk) ↑, IL-6 ↔	[29]

Legend: ↓, significant decrease; ↑, significant increase; ↔, no significant changes; d, days; F, female; HDL, high-density lipoproteins; IBS, irritable bowel syndrome; IFN-γ, interferon gamma; IL-1ra, interleukin 1 receptor antagonist; IL-6, interleukin 6; IL-8, interleukin 8; IL-10, interleukin 10; IL-12, interleukin 12; IL-17, interleukin 17; IL-1β, interleukin 1 beta; IL-4, interleukin 4; IP-10, interferon gamma-induced protein 10; MCP-1, monocyte chemoattractant protein 1; MIP-1β, macrophage inflammatory protein 1 beta; n.d., not declared; T, treatment; TNF-α, tumor necrosis factor alpha; wk, weeks.

Among the six studies that reported changes in cytokine levels, four involved whole grain interventions. One study on healthy participants showed a decrease in IL-6 levels after a 4-week combination of 60 g/day of barley and brown rice, but not after consuming barley or brown rice separately [27]. The other three studies were focused on overweight/obese participants. Specifically, Kopf and colleagues [26] observed a reduction in TNF-α but no changes in IL-6 levels after 6-week consumption of three servings/day of whole grains compared to refined grains. Vitaglione and coworkers [29] reported a decrease in TNF-α and an increase in IL-10 in the whole-grain wheat group (70 g/d), while IL-6 levels remained unchanged. Then, a reduction in IL-6 and IL-1β, but not in TNF-α, was observed after 8 weeks of consuming 75 g/day of whole grain products among overweight/obese individuals at risk of metabolic syndrome [28].

Changes in cytokine levels were also demonstrated after a 6-week intervention with baked products made with ancient or modern wheats (the latter as control) in participants with moderate irritable bowel syndrome, with reductions in IL-4, IL-6, IL-17, IFN-γ, and MCP-1, but not IL-8, IL-10, IL-12, MIP-1β, TNF-α, or IP-10 [20]. A similar trend was observed in an 8-week study providing the same portions of Khorasan wheat (Kamut^®^, Great Falls, MT, USA) or semi-whole grain wheat (control) to healthy participants, where reductions in IL-6, IL-12, MCP-1, MIP-1β, and TNF-α were observed, though no changes were seen in IL-1ra, IL-4, IL-8, IL-10, IL-17, or IP-10, although it is noteworthy that reductions in MCP-1 and MIP-1β levels were also seen in the control group consuming semi-whole wheat products [21].

#### 3.1.2. Fruits and Vegetables

Among the nine studies evaluating fruits and vegetables (Table 2), only two studies focused on vegetables, namely broccoli [30], cabbage, and cucumbers [31]. Four studies investigated interventions with fruits, specifically grapes [32], strawberries [33], cherries [34], and bilberries [35], while three studies included different fruits and vegetables [26,36,37]. These interventions involved 416 participants overall. Six out of seven studies reported variations in cytokine levels.

Consuming fermented and pickled cabbage and cucumber did not impact TNF-α levels in healthy women [31]. In contrast, a reduction in IL-6 levels was observed following the consumption of 30 g/day of raw broccoli sprouts for 70 days in healthy overweight individuals. This reduction persisted for 90 days after the end of the treatment [30]. Two of the studies involving fruits compared freeze-dried fruits to matched placebo powders. In one of them, 46 g/day of freeze-dried grape powder, given for 4 weeks, resulted in decreased IL-10 and adiponectin levels in overweight/obese men with metabolic syndrome and dyslipidemia. However, there was no effect on TNF-α, IL-6, or IL-8 levels [32]. In another study, 50 g twice/day of freeze-dried strawberries for 12 weeks led to a reduction in TNF-α levels in obese adults with knee osteoarthritis, while IL-19 levels remained unchanged [33].

Kelley and colleagues found that 28-day consumption of 280 g/day of cherries led to a decrease in IL-18 levels and an increase in IL-1ra in adults with modestly elevated blood C-reactive protein, while TNF-α levels remained unchanged [34]. A study involving an 8-week intervention with bilberries in overweight/obese individuals with metabolic syndrome did not observe differences in IL-6, IL-12, or adiponectin at the end of the study. However, a reduction in IL-6 and IL-12 after 12 weeks from the beginning of the intervention (4 weeks of follow-up) was detected [35].

All studies examining the increase in fruit and vegetable portions focused on individuals who do not consume the suggested amounts of these foods. A study involving a gradual increase in high- or low-flavonoid fruits and vegetable consumption did not lead to variations in IL-6 and TNF-α [36], while the consumption of five servings/day for 16 weeks led to a reduction in TNF-related apoptosis-inducing ligand (TRAIL), TNF-related activation-induced cytokine (TRANCE), and C-X3-C motif chemokine ligand-1 (CX3CL1), but not of IL-6, TNF-α, MIP-1α, MIP-1β, or IL-18, in older adults [37]. Additionally, the study by Kopf and colleagues previously mentioned in Section 3.1.1 included an intervention arm providing three servings/day of fruits and vegetables to overweight/obese participants. This study observed a significant reduction in IL-6 levels in the treatment group after 6 weeks, while TNF-α levels remained stable [26].

**Table 2:** **Summary of randomized controlled trials investigating the effects of fruits and vegetables on circulating cytokines**. Each study includes details of the design, duration, treatment, dose, study population, and reported outcomes. Results are presented as changes within groups and between groups.

Study Design (Duration)	Treatment	Serving Size	Study Population	Population Number (F)	Results Within Groups	Results Between Groups	Reference
Randomized, controlled, crossover, double-blind (4 wk × 2)	T: freeze-dried grape powder C: macronutrient and caloric matched powder	46 g/d	Men; overweight/obese; metabolic syndrome. With dyslipidemia vs. no dyslipidemia	24 (0)	(n.d.)	T—Dyslipidemic vs. non-dyslipidemic: IL-10, adiponectin ↓; TNF-α, IL-6, IL-8 ↔	[32]
Randomized, controlled, crossover, double-blind (12 wk × 2)	T: freeze-dried strawberries C: macronutrient and caloric matched powder	50 g twice/d	Obese; knee osteoarthritis	17 (13)	-	T vs. C: TNF-α ↓, IL-19 ↔	[33]
Randomized, controlled, parallel, double-blind (6 wk)	T1: fermented cabbage and cucumber T2: pickled cabbage and cucumber C: usual diet	0.5 cups/d, equivalent to 100 g cabbage or 80 g cucumbers	Women, healthy	31 (31)	T1: TNF-α ↔ T2: TNF-α ↔ C: TNF-α ↔	T1 vs. T2 vs. C: TNF-α ↔	[31]
Self-controlled (28 d)	T: cherries	280 g/d	Normal weight/overweight; generally healthy with modestly elevated C-reactive protein	18 (16)	T: TNF-α ↔, IL-18 ↓ IL-1ra ↑	(n.d.)	[34]
Randomized, controlled, parallel (8 wk)	T: puree and dried bilberries C: usual diet	T: 200 g of bilberry puree and 40 g of dried bilberries	Overweight/obese; metabolic syndrome	34 (n.d.)	T: IL-6, IL-12 ↓ adiponectin ↔ (12th wk vs. 8th wk)	T vs. C: IL-6, IL-12 adiponectin ↔ (8th wk)	[35]
Randomized, controlled, parallel (6 wk)	T: fruits and vegetables C: refined grains	3 servings/d	Overweight, obese, Low intakes of fruit, vegetables, and whole grains	32 (19)	T: TNF-α ↔; IL-6 ↓ C: TNF-α, IL-6 ↔	(n.d.)	[26]
Self-controlled with follow-up (70 d) + follow-up at 90 and 160 d (=20 and 90 d no broccoli)	T: raw broccoli sprouts	30 g/d	Overweight; healthy	40 (19)	70 d: IL-6 ↓ 90 d: IL-6 ↓ 160 d: IL-6 ↓	(n.d.)	[30]
Randomized, controlled, parallel, open label (6 wk × 3)	T1: high flavonoid fruits and vegetables T2: low flavonoid fruits and vegetables C: usual diet	80 g/d × 2 (1st wk), ×4 (2nd wk), ×6 (3rd wk)	Risk of CVD, <4.4 servings of fruits and vegetables/d	154 (94)	(n.d.)	T1 vs. T2 vs. C: IL-6, TNF-α ↔	[36]
Randomized, controlled, parallel (16 wk)	T: fruits and vegetables/day C: usual diet	5 servings/d	Older adults (65–70 years); <5 fruits and vegetables servings/d	66 (36)	T: TRAIL, TRANCE, CX3CL1 ↓ IL-6, TNF-α, MIP-1α, MIP-1β, IL-18 ↔ C: TRAIL, TRANCE, CX3CL1, IL-6, TNF-α, MIP-1α, MIP-1β, IL-18 ↔	(n.d.)	[37]

Legend: ↓, significant decrease; ↑, significant increase; ↔, no significant changes; C, control; CX3CL1, C-X3-C motif chemokine ligand-1; d, days; F, female; IL-1ra, Interleukin 1 Receptor Antagonist, IL-6, interleukin 6; IL-8, interleukin 8; IL-10, interleukin 10; IL-12, interleukin 12; IL-18, interleukin 18; IL-19, interleukin 19; M, male; MIP-1α, macrophage inflammatory protein-1α; MIP-1β, macrophage inflammatory protein-1β; n.d., not declared; TRAIL, TNF-related apoptosis-inducing ligand; TRANCE, TNF-related activation-induced cytokine; T, treatment; TNF-α, tumor necrosis factor alpha; wk, weeks.

#### 3.1.3. Oils and Fats

Seven studies, reported in Table 3, focused on the effects of consumption of different oils and fats on inflammatory markers. Across all studies in this category, a total of 304 participants were examined. Two out of the six studies involving oils compared differently processed olive oils (extra virgin [38] or virgin [39] vs. refined), two compared canola and olive oil [40,41], one compared linseed oil to safflower oil [42], and one, flaxseed oil to high-oleic sunflower oil [43]. One study evaluated different types of margarine [44]. All the studies involved participants with pathologies, risk factors, and/or who were overweight/obesity. Four studies out of seven showed an impact of the treatment on cytokines levels.

**Table 3:** **Summary of randomized controlled trials investigating the effects of oils and fats on circulating cytokines**. Each study includes details of the design, duration, treatment, dose, study population, and reported outcomes. Results are presented as changes within groups and between groups.

Study Design (Duration)	Treatment	Serving Size	Study Population	Population Number (F)	Results Within Groups	Results Between Groups	Reference
Randomized, crossover, placebo-controlled, double-blind (3 wk × 2)	T: virgin olive oil C: refined olive oil	50 mL/d	Coronary heart disease	28	(n.d.)	T vs. C: IL-6 ↓	[38]
Randomized, controlled, crossover, double-blind (4 wk × 3)	T1: margarine with sterols from rapeseed T2: margarine with sterols from tall C: non-sterol margarine	25 g/d	Normal weight/overweight; hypercholesterolemia	51 (n.d.)	(n.d.)	T1 vs. T2 vs. C: TNF-α ↔	[44]
Randomized, controlled, parallel, double blind (12 wk)	T: flaxseed oil C: high-oleic sunflower oil	10 g/d	Older adults (60 ± 8 y.o.); overweight/obese; healthy with high-normal blood pressure or mild (stage I) hypertension	59 (19)	T: TNF-α ↓ IL-6, IL-8, MCP-1 ↔ C: TNF-α, IL-6, IL-8, MCP-1 ↔	T vs. C: TNF-α, IL-6, IL-8, MCP-1 ↔	[43]
Randomized, controlled, parallel, open label (6 wk)	T1: refined olive oil T2: canola oil	25 mL/d	Men and post-menopausal women with at least one cardiovascular risk factor (hypertension, dyslipidemia, diabetes)	42 (4)	-(n.d.)	T1 vs. T2: IL-6 ↓	[40]
Randomized, two arm, parallel (4 wk)	T1: rapeseed/canola oil T2: olive oil	50 g/d	Men; overweight/obese	18 (0)	T1: IL-6, MCP-1 ↔ T2: IL-6, MCP-1 ↔	T1 vs. T2: IL-6, MCP-1 ↔	[41]
Randomized, two arm, parallel (12 wk)	T1: linseed oil (ALA) T2: safflower oil (LA)	15 mL/d	Men; dyslipidemia	76 (0)	T1: IL-6 ↓ T2: IL-6 ↔	T1 vs. T2: IL-6 ↓	[42]
Randomized, controlled, parallel double blind (3 wk)	T1: extra virgin olive oil T2: refined olive oil	50 mL/d	Women, fibromyalgia	30 (30)	T1: IL-6, IL-10 ↔ T2: IL-6, IL-10 ↔	T1 vs. T2: IL-6, IL-10 ↔	[39]

Legend: ↓, significant decrease, ↔, no significant changes; ALA, alpha-linolenic acid; C, control; d, days; F, female; IL-6, interleukin 6; IL-8, interleukin 8; IL-10, interleukin 10; LA, linoleic acid; MCP-1, monocyte chemoattractant protein 1; n.d., not declared; T, treatment; TNF-α, tumor necrosis factor alpha; wk, weeks.

In a 12-week intervention, men with dyslipidemia consumed 15 mL/day of linseed oil (high alpha-linolenic acid) or safflower oil (high linoleic acid). Lower IL-6 following linseed oil consumption was observed both within and between treatments [42]. In another study, participants with coronary heart disease consumed 50 mL/day of virgin olive oil or refined olive oil over 3 weeks. The results showed significantly lower IL-6 levels in the group consuming virgin olive oil [38]. The same cytokine was lowered after 25 mL/day of canola oil compared to refined olive oil for 6 weeks in men and post-menopausal women with cardiovascular risk factors [40]. In a 12-week study, overweight or obese older adults that were pre-hypertensive or had mild (stage I) hypertension received either flaxseed oil or high-oleic sunflower oil (10 g/day). While the high-oleic sunflower oil group showed no changes in TNF-α, IL-6, IL-8, or monocyte chemoattractant protein-1 (MCP-1), the flaxseed oil group showed a decrease limited to TNF-α levels. However, comparison between the two groups did not reveal significant differences in any of these markers [43]. On the contrary, two studies did not register any change in cytokine levels, neither for extra virgin or refined olive oil in women with fibromyalgia [39], nor for rapeseed/canola or olive oil in overweight/obese men [41]. The consumption of three types of margarine with different sterol contents did not influence TNF-α levels in non-obese participants with hypercholesterolemia [44].

#### 3.1.4. Seeds and Nuts

Among the seven studies on seeds and nuts, summarized in Table 4, five studies focused on seeds, specifically sesame seeds [45] and flaxseeds [46,47,48,49], while two involved nuts, such as walnuts [50] and hazelnuts [51]. The studies involved a total of 428 participants. Only two studies out of seven showed a modulation in cytokines.

**Table 4:** **Summary of randomized controlled trials investigating the effects of seeds and nuts on** circulating cytokines. Each study includes details of the design, duration, treatment, dose, study population, and reported outcomes. Results are presented as changes within groups and between groups.

Study Design (Duration)	Treatment	Serving Size	Study Population	Population Number (F)	Results Within Groups	Results Between Groups	Reference
Randomized, controlled, crossover, single-blind (4 wk × 3)	T: walnuts C: usual diet—avoid nuts or fish	Walnuts: 6 d/wk	Adults; normal to mildly hyperlipidemic	25 (n.d.)	(n.d.)	T1 vs. T2 vs. C: IL-1β, IL-6, TNF-α ↔	[50]
Randomized, controlled, parallel (2 months)	T: sesame seeds powder C: placebo powder	40 g/d	Knee osteoarthritis	45 (n.d.)	T: IL-6 ↓ C: IL-6 ↓	T vs. C: IL-6 ↓	[45]
Randomized, controlled, crossover, open label (12 wk × 3)	T1: lower portion of ground flaxseed T2: higher portion of ground flaxseed C: usual diet—no flaxseed	T1: 13 g/d T2: 26 g/d	Men: overweight/obese. Women: post-menopausal, pre-diabetic.	25 (14)	(n.d.)	T1 vs. T2 vs. C: IL-6, adiponectin ↔	[48]
Randomized, controlled, parallel (12 wk)	T: dietary recommendations + flaxseed C: dietary recommendation	30 g/d	Coronary heart disease. Non-obese men and post-menopausal women who have been planned for percutaneous coronary intervention.	44 (7)	(n.d.)	T vs. C: IL-6, TNF-α ↓	[46]
Randomized, controlled, crossover (12 wk × 2)	T: flaxseed C: wheat bran	40 g/d	Overweight/obese, glucose intolerant, hypertensive, with a family history of diabetes	9 (5)	T: TNF-α, IL-6 ↔ C: TNF-α, IL-6 ↔	T vs. C: TNF-α, IL-6 ↔	[47]
Randomized, controlled, parallel, single-blind (12 wk)	Hazelnuts: T1: lower serving size T2: higher serving size C: usual diet—avoid nuts	T1: 30 g/d T2: 60 g/d	Adults; overweight/obese; healthy	107 (61)	T1: IL-6 ↔ T2: IL-6 ↔ C: IL-6 ↔	T1 vs. T2 vs. C: IL-6 ↔	[51]
Randomized, controlled, parallel, single-blind (12 wk)	T: lifestyle counseling + bread with flaxseed C: lifestyle counseling + bread	30 g flaxseed/d in 100 g bread	Participants with at least 2 Metabolic syndrome components	173 (76)	(n.d.)	T vs. C: IL-6, IL-18, TNF-α, MCP-1 ↔	[49]

Legend: ↓, significant decrease, ↔, no significant changes; C, control; d, days; F, female; IL-1β, interleukin 1 beta; IL-6, interleukin 6; IL-18, interleukin 18; MCP-1, monocyte chemoattractant protein 1; n.d., not declared; T, treatment; TNF-α, tumor necrosis factor alpha; wk, weeks.

A 2-month study examined the effects of sesame seed powder (40 g/day) compared to a placebo powder in individuals with knee osteoarthritis, obtaining a significant decrease in IL-6 levels in both the treatment and control groups, but with the sesame seed group showing significantly lower IL-6 with respect to the control group [45]. Flaxseeds were indeed the most investigated item among this group, generally with no significant results. In a 12-week study, non-obese men and post-menopausal women with coronary heart disease were provided with 30 g/day of flaxseed: the intervention group showed lower IL-6 and TNF-α levels than the control group [46].

Conversely, flaxseed consumption did not exert modulation of cytokines in overweight or obese participants described as “glucose intolerant”, hypertensive, and who had a family history of diabetes [47], overweight/obese men and pre-diabetic post-menopausal women [48], or in participants with at least two metabolic syndrome components [49].

In the two studies that involved nuts, modulation of cytokines was observed in neither walnuts, which were tested in healthy to mildly hyperlipidemic participants [50], nor hazelnuts, which were tested in healthy overweight or obese participants [51].

#### 3.1.5. Fish

The seven studies shown in Table 5 concerned fish: two studies compared different types of fishes, specifically oily vs. lean fish [52] and different species of oily fishes [53], two focused on differently fed fish such as salmon [54] and gilthead sea bream [55], one on different servings of salmon [56], one on sea bass [57], and one compared fish and nut consumption [50]. The total number of participants included was 349. Overall, five out of seven studies showed a reduction in cytokine production.

**Table 5:** **Summary of randomized controlled trials investigating the effects of fish on** circulating cytokines. Each study includes details of the design, duration, treatment, dose, study population, and reported outcomes. Results are presented as changes within groups and between groups.

Study Design (Duration)	Treatment	Serving Size	Study Population	Population Number (F)	Results Within Groups	Results Between Groups	Reference
Randomized, controlled, crossover, single-blind (4 wk × 3)	T: fish C: usual diet—avoid nuts or fish	Fish: two servings/twice wk	Adults; normal to mildly hyperlipidemic	25 (n.d.)	(n.d.)	T1 vs. T2 vs. C: IL-1β, IL-6, TNF-α ↔	[50]
Randomized, crossover (4 wk × 3)	Salmon filets serving size: T1: lower T2: intermediate T3: higher	T1: 90 g twice/wk. T2: 180 g twice/wk. T3: 270 g twice/wk.	Overweight/obese; healthy; low fish intake	19 (11)	T1: IL-6 ↔ T2: IL-6 ↔ T3: IL-6 ↔	T1 vs. T2 vs. T3: IL-6 ↔	[56]
Randomized, parallel, double-blind (6 wk)	Atlantic salmon: T1: fed on 100% fish oil; containing higher *n*-3 PUFAs T2: fed on 50% fish oil and 50% rapeseed oil; containing intermediate *n*-3 PUFAs T3: fed on 100% rapeseed oil low *n*-3 PUFAs	700 g per wk	Coronary heart disease	58 (8)	T1: TNF-α, IL-6, IL-10 ↔ T2: TNF-α ↓ IL-6 ↔ IL-10 ↑ T3: TNF-α, IL-6, IL-10 ↔	T1 vs. T2: IL-6 ↓; TNF-α, IL-10 ↔ T1 vs. T3 IL-6 ↓; TNF-α, IL-10 ↔	[54]
Self-controlled (10 wk)	Sea bass filets	800 g/wk	Dyslipidemia	9 (5)	IL-6, IL-8 ↓	(n.d.)	[57]
Randomized, crossover, single-blind (10 wk × 2)	Group A: T1: gilthead sea bream filets, fed with 100% fishmeal T2: gilthead sea bream filets, fed with fishmeal + plant protein	630 g/wk	Normal weight, overweight; healthy	20 (8)	Group A—T1: IL-6, IL-8 ↓; TNF-α, IL-10 ↔ Group A—T2: IL-6, IL-8, TNF-α, IL-10 ↔	-	[55]
	Group B: T1: Gilthead sea bream filets, fed with fishmeal + plant protein T2: Gilthead sea bream filets, fed with 100% fishmeal				Group B—T1: IL-6 ↓; IL-8, TNF-α, IL-10 ↔ Group B—T2: IL-6, IL-8, TNF-α, IL-10 ↔	-	
Randomized, controlled, parallel (8 wk)	T1: oily fish T2: freshwater fish C: pork/chicken/beef	T1:100 g salmon/lunch, 5 times/wk T2: 100 g fish/lunch; hairtail (2 times/wk), carp (1 time/wk), or grass carp (1 time/wk) T3: 100 g meat/lunch; pork (3 times/wk), chicken (1 time/wk), or beef (1 time/wk)	Male; overweight/obese, dyslipidemia	92 (0)	T1: IL-6 ↓ T2: IL-6 ↔ T3: IL-6 ↔	T1 vs. T2 vs. T3: IL-6 ↔	[52]
Randomized, parallel, controlled (8 wk)	T1: Norwegian salmon T2: herring T3: pompano C: pork/chicken/beef/lean fish	T1, T2, T3: lunch meal with 80 g/d filet of fish, 5 d/wk C: 80 g/d of pork (2 d/wk), beef (1 d/wk), chicken (1 d/wk) or lean fish meals (1 d/wk)	Middle-aged and elderly women, dyslipidemia	126 (126)	T1: TNF-α, IL-6 ↓; adiponectin ↑ T2: TNF-α ↓, IL-6 ↔; adiponectin ↑ T3: TNF-α, IL-6, adiponectin ↔ C: TNF-α, IL-6, adiponectin ↔	TNF-α, IL-6, adiponectin ↔	[53]

Legend: ↓, significant decrease; ↑, significant increase; ↔, no significant changes; C, control; F, female; IL-1β, interleukin 1 beta; IL-6, interleukin 6; IL-8, interleukin 8; IL-10, interleukin 10; n.d., not declared; T, treatment; TNF-α, tumor necrosis factor alpha; d, days; wk, weeks.

Among the two studies that compared fishes, one of them focused on oily vs. lean fish. Overweight/obese males with dyslipidemia were divided into three groups, each consuming either 100 g of salmon as oily fish; hairtail, carp, and grass carp as freshwater fish; or various meats (control) for five times/week for 8 weeks. Results indicated that IL-6 levels decreased in the group consuming oily fish, while there were no significant changes for the freshwater fish or meat groups and no significant changes between groups [52]. In a study comparing three species of oily fishes, middle-aged and elderly women with dyslipidemia consumed 80 g/day of either Norwegian salmon, herring, or pompano filet five times a week, while the control group consumed 80 g/day of pork, beef, chicken, or lean fish. Results showed a decrease in TNF-α and IL-6 levels, along with an increase in adiponectin levels, in participants consuming Norwegian salmon and herring. In contrast, the group consuming pompano, as well as the control group, showed no significant changes in TNF-α, IL-6, or adiponectin levels. Additionally, no significant differences were observed between the treatment groups [53].

Among two studies comparing differently fed fish, one focused on Atlantic salmon fed with oils containing varying levels of *n*-3 PUFAs: higher, intermediate, and lower. Participants with coronary heart disease consumed 700 g per week of one type of salmon for 6 weeks. While the higher and lower *n*-3 PUFA salmons induced no changes in TNF-α, IL-6, or IL-10 levels, the intermediate group experienced a decrease in TNF-α and an increase in IL-10. Comparing groups, the higher PUFA salmon group showed a greater reduction in IL-6 than the others, but no differences in IL-10 or TNF-α [54]. In a study by Sofi et al. [55], gilthead sea bream filets fed with 100% fishmeal were compared to filets fed with a mix of fishmeal and plant protein. Healthy participants consumed the filets in two different orders for 10 weeks each. In one sequence, IL-6 and IL-8 levels decreased after consuming 100% fishmeal-fed filets, with no changes in TNF-α or IL-10, while the mixed diet showed no significant effects. In the reverse order, no changes were observed with the mixed diet, but IL-6 levels decreased after consuming the 100% fishmeal diet.

Consuming sea bass for ten weeks induced a reduction in IL-6 and IL-8 in dyslipidemic participants [57]. When different populations of healthy obese and overweight individuals consumed different servings of salmon filets, no significant differences were observed in IL-6 levels either within groups or between them, regardless of the salmon serving size [56].

The study by Chiang and colleagues [50] (previously discussed in Section 3.1.4) also included an arm in which participants consumed fish. No significant differences were observed in IL-1β, IL-6, and TNF-α between groups.

#### 3.1.6. Dairy

A total of six studies investigated the effect of dairy products (Table 6): three of them focused on yogurt [58,59,60], one on cheese [61] and two on a combination of dairy products [62]. A total of 384 individuals participated in the studies assessing this food group. Among the six dairy-based interventions, cytokine levels decreased in three of the six cases, while one study on yogurt reported an increase in cytokines, specifically among healthy participants [59]. In the study by Pei and colleagues, the effects of low-fat yogurt consumption over a 9-week period were compared to soy pudding (control) in pre-menopausal women. Low-fat yogurt consumption led to a decrease in TNF-α levels, but not in IL-6, while the control group showed no changes. However, there were no significant differences in TNF-α or IL-6 between the yogurt and soy pudding groups [58]. In a study involving non-diabetic older adults, participants were asked to consume 120 mL/day of yogurt or milk (control) over a 12-week period. No significant changes in TNF-α or IFN-γ levels were observed within the yogurt group, although IL-12 levels increased. In the control group, which consumed milk, cytokine levels remained unchanged. When comparing the two groups, there were no differences in TNF-α or IL-12 levels, but INF-γ levels were higher in the yogurt group [59]. Conversely, consumption of probiotic yogurt daily did not influence TNF-α and IL-6 in individuals with nonalcoholic minimal hepatic encephalopathy cirrhosis [60].

**Table 6:** **Summary of randomized controlled trials investigating the effects of dairy foods on circulating cytokines**. Each study includes details of the design, duration, treatment, dose, study population, and reported outcomes. Results are presented as changes within groups and between groups.

Study Design (Duration)	Treatment	Serving Size	Study Population	Population Number (F)	Results Within Groups	Results Between Groups	Reference
Randomized, controlled, parallel, single-blind (60 d)	T: yogurt C: usual diet	12 ounces/d	Non-alcoholic minimal hepatic encephalopathy cirrhosis	22 (n.d.)	T: TNF-α, IL-6 ↔ C: TNF-α, IL-6 ↔	(n.d.)	[60]
Randomized, controlled, crossover (6 wk × 2)	T: low-fat dairy products C: carb-based products	T: 283 g 1% fat milk, 170 g nonfat yogurt, 113 g 2% fat cheese C: 43 g granola bar, 340 g 100% juice	Metabolic syndrome; Low intake of dairy	33 (21)	(n.d.)	T vs. C (women): TNF-α, MCP-1 ↓; leptin, adiponectin ↔ T vs. C (men): TNF-α, MCP-1, leptin, adiponectin ↔ T vs. C (all): TNF-α, MCP-1, leptin, adiponectin ↔	[62]
Randomized, controlled, parallel, open label (12 wk)	T: yogurt C: milk	120 mL/d	Older adults (>60 y.o.); non-diabetic, healthy	152 (107)	T: TNF-α, IFN-γ ↔ IL-12 ↑ C: T: TNF-α, IFN-γ, IL-12 ↔	T vs. C: TNF-α, IL-12 ↔ IFN-γ ↑	[59]
Randomized, controlled, parallel, open label (9 wk)	T: low-fat yogurt C: soy pudding	T: 339 g/d C: 324 g/d	Pre-menopausal women; non-obese vs. obese.	120 (120)	T—non-obese: TNF-α ↓; IL-6 ↔ T—obese: TNF-α ↓; IL-6 ↔ C—non-obese: TNF-α, IL-6 ↔ C—obese: TNF-α, IL-6 ↔	TNF-α, IL-6 ↔	[58]
Controlled, crossover (one sequence) (10 wk × 2)	T: Pecorino Toscano cheese C: cow cheese	200 g/wk, to be divided into three portions (3 times/wk)	Healthy	10 (6)	T: TNF-α, Il-6, IL-8 ↓; IL-10, IL-12 ↔ C: TNF-α, Il-6, Il-8, IL-10, IL-12 ↔	T vs. C: TNF-α, Il-6, Il-8 ↓; IL-10, IL-12 ↔	[61]
Randomized, controlled, crossover (4 wk × 3)	T: low-fat dairy (milk, yogurt, custard and cheese) C: avoid red meat + eat < 1 servings of dairy/day	T: 4–6 servings/day of 250 g milk, 200 g yogurt, 40 g hard cheese, or 120 g ricotta cheese	Overweight and obese	47 (29)	(n.d.)	T1 vs. T2 vs. C: TNF-α ↔	[63]

Legend: ↓, significant decrease; ↑, significant increase; ↔, no significant changes; d, days; F, female; n.d., not declared; IFN-γ, interferon gamma; IL-6, interleukin 6; IL-8, interleukin 8; IL-10, interleukin 10; IL-12, interleukin 12; MCP-1, monocyte chemoattractant protein 1; n.d., not declared; TNF-α, tumor necrosis factor alpha; wk, weeks.

As regards cheese, in a study by Sofi and coworkers [61], healthy individuals consumed 200 g of either pecorino toscano cheese or cow cheese (control) per week for 10 weeks. The results showed a significant decrease in TNF-α, IL-6, and IL-8 levels in the pecorino group, which was not observed in the control group; comparing the groups, these results were confirmed.

Two different studies focused on low-fat dairy consumption. A 6-week trial compared the effects of dairy products with those of carbohydrate-based products in individuals with metabolic syndrome and low dairy intake. While no significant differences were observed between the two groups for all participants and for men in particular, in women, the results showed lower levels of TNF-α and MCP-1 in the dairy-treated group, while leptin and adiponectin levels remained comparable [62]. No differences in TNF-α levels in overweight/obese participants were found following low-fat dairy or red meat consumption with respect both to the control group (no meat, low dairy) and to each other [63].

#### 3.1.7. Miscellaneous Solid Foods

The miscellaneous group includes a total of six studies, as presented in Table 7. Among them, three studies provided subjects with legumes [64], red meat [63], bison meat [65], honey [66], and a “functional” soup [67]. Moreover, one included a combined intervention with flaxseeds, olive oil, and seeds [68]. The interventions involved a total of 255 participants. Overall, in the miscellaneous group, only two studies of six displayed a reduction in cytokine circulating levels.

**Table 7:** **Summary of randomized controlled trials investigating the effects of miscellaneous foods on circulating cytokines**. Each study includes details of the design, duration, treatment, dose, study population, and reported outcomes. Results are presented as changes within groups and between groups.

Study Design (Duration)	Treatment	Serving Size	Study Population	Population Number (F)	Results Within Groups	Results Between Groups	Reference
Randomized, parallel, controlled, open label (12 wk)	T: honey C: usual diet	20 g/d	Chronic smokers	62 (n.d.)	T: TNF-α ↑ IL-6 ↔ C: TNF-α, IL-6 ↔	(n.d.)	[66]
Randomized, controlled, parallel, double-blind (7 d)	T: soup containing functional foods C: barley soup	T: 30 g of mixed functional foods C: barley soup	COVID-19	60 (32)	T: IL-1β, IL-6, IL-17, TNF-α, IFN-γ ↓ IL-10 ↑ C: IL-1β, IL-6, IL-17, TNF-α, IFN-γ ↓ IL-10 ↑	T vs. C: IL-1β, IL-6, IL-17, TNF-α ↓ IFN-γ ↔ IL-10 ↑	[67]
Randomized, controlled, parallel, open label (30 months)	T: dietary recommendations + flaxseed + olive oil C: dietary recommendations	30 g flaxseed/d 25 mL olive oil/d	Non-obese, CHD-1 month after coronary angioplasty	50 (11)	T: IL-6, TNF-α, MCP-1 ↓; IL-10 ↔ C: IL-6, TNF-α, MCP-1, IL-10 ↔	T vs. C: TNF-α, IL-6, MCP-1↓; IL-10 ↔	[68]
Randomized, crossover, two arm, double-blind (7 wk × 2)	T1: bison meat T2: beef	340 g/d, 6 d/wk	Male; healthy	10 (0)	T: IL-10, IL-6, TNF-α ↔ C: IL-10, IL-6, TNF-α ↔	T vs. C: IL-6 ↓, IL-10, TNF-α ↔	[65]
Randomized, controlled, crossover, open label (6 wk × 2).	T: legumes (pinto beans and brown lentils) C: usual diet	65 g (raw), 4 times/wk	Overweight/centrally obese At risk for diabetes	26 (14)	T: TNF-α, IL-6, adiponectin ↔ C: TNF-α, IL-6, adiponectin ↔	T vs. C: TNF-α, IL-6, adiponectin ↔	[64]
Randomized, controlled, crossover (4 wk × 3)	T: red meat + eat <1 servings of dairy/d C: avoid red meat + eat < 1 servings of dairy/d	>200 g, 6 times/wk	Overweight and obese	47 (29)	(n.d.)	T1 vs. T2 vs. C: TNF-α ↔	[63]

Legend: ↓, significant decrease; ↑, significant increase; ↔, no significant changes; C, control. d, days; F, female; n.d. not declared; IFN-γ, interferon gamma; IL-6, interleukin 6; IL-10, interleukin 10; IL-17, interleukin 17; IL-1β, interleukin 1 beta; MCP-1, monocyte chemoattractant protein 1; macrophage inflammatory protein 1 beta; n.d., not declared; T, treatment; TNF-α, tumor necrosis factor alpha; wk, weeks.

The effects of legume consumption, such as the consumption of pinto beans and brown lentils, on overweight and obese individuals at risk for diabetes were investigated in a 6-week trial. No changes were observed in TNF-α, IL-6, or adiponectin levels either within or between groups [64].

In a study examining meat products, male participants consumed 340 g/day of either bison or beef six days a week for 7 weeks. The study found no significant changes in IL-10, IL-6, or TNF-α within the groups. However, when comparing the two groups, the beef group showed an increase in the cytokine IL-6 [65]. In a study previously cited in the dairy section, red meat was evaluated as a food group and compared with dairy; no differences between groups in TNF-α were observed in overweight/obese participants [63].

When the impact of 20 g/day honey consumption was evaluated in chronic smokers over 12 weeks, an increase in the levels of pro-inflammatory TNF-α, but not IL-6, was detected [66].

Moreover, a 30-month study evaluated the effect of 30 g flaxseed/day and 25 mL olive oil/day in non-obese individuals with coronary heart disease. The treatment group showed reductions in IL-6, TNF-α, and MCP-1 with respect to the baseline and control group, while IL-10 remained stable [68].

Participants with COVID-19 were given either a soup containing functional foods (including various ingredients such as wheat, rice, peas, apples, and various herbs) or a barley soup (control group) for 7 days. The group consuming the functional food soup showed significantly lower levels of IL-1β, IL-6, IL-17, TNF-α, and IFN-γ, along with higher IL-10, both when compared to their baseline and the control group [67].

### 3.2. Beverages

#### 3.2.1. Fruit Juices

The effects of fruit juices were investigated in a total of 19 studies, outlined in Table 8. Among these, eight studies focused on berries, three of which specifically focused on cranberries [69,70,71]. The other studies investigated bilberry [72] and chokeberry [73], goji berries [74] the Chinese bayberry [75], and the Amazonian camu-camu [76]. In addition, studies investigated cherries [77,78,79,80], apples [81,82], tomatoes [83,84], red oranges [85], plums [86], and pomegranates [87]. Altogether, the studies involving fruit juices enrolled 841 participants. A reduction in cytokines was observed in nine out of nineteen studies, mainly in participants affected by pathologies or with health risk factors, while three studies reported increases, all of them involving healthy participants.

**Table 8:** **Summary of randomized controlled trials investigating the effects of fruit** and vegetable juices. Each study includes details of the design, duration, treatment, dose, study population, and reported outcomes. Results are presented as changes within groups and between groups.

Study Design (Duration)	Treatment	Dose	Study Population	Study Population Number (F)	Results Within Groups	Results Between Groups	References
Randomized, controlled, parallel, double-blind (30 d)	T: goji berry juice C: placebo	60 mL, twice/d	Older adults (55–72 y.o.)	60 (n.d.)	T: IL-2 ↑; IL-4 ↔ C IL-2, IL-4 ↔	T vs. C: IL-2 ↑; IL-4 ↔	[74]
Randomized, controlled, parallel (30 d)	T: dark sweet cherry juice supplemented with dark sweet cherry powder C: placebo juice	200 mL twice/d	Obese	40 (24)	T: IL-1ra, IL-18, TNF-α, RANTES/CCL5, IL-6, IL-10, MCP-1, IFN-γ, IL-1β ↔ C: IL-18, TNF-α, RANTES/CCL5, IL-6, MCP-1, IFN-γ, IL-1β ↔; IL-1ra ↓; IL-10↑	T vs. C: IL-1ra, IL-18, TNF-α, RANTES/CCL5, IL-6, MCP-1 ↔; IL-10, IFN-γ, IL-1β ↓	[77]
Randomized, controlled, parallel, blinded (4 wk)	T: polyphenol-rich apple juice C: placebo	750 mL/d	Men; overweight/obese	68 (0)	T: TNF-α, IL-6 ↔ C: TNF-α, IL-6 ↔	T vs. C: TNF-α, IL-6 ↔	[81]
Randomized, controlled, parallel, double-blind (8 wk)	T: low energy cranberry juice C: placebo	480 mL/d	Women; 3/5 criteria for metabolic syndrome	31 (31)	(n.d.)	T vs. C: IL-6 ↔	[70]
Randomized, controlled, crossover 2 × 2, single-blind (1 wk × 2)	T: red orange juice C: placebo	250 mL, twice/d	Nondiabetic, increased cardiovascular risk, >2 criteria for metabolic syndrome; overweight/obese. Control: Healthy, non-obese	Cardiovascular risk: 19 Healthy control: 12 (7) TOT 31 (16)	T—Cardiovascular risk: TNF-α, IL-6 ↓ C—Cardiovascular risk: TNF-α, IL-6 ↔	(n.d.)	[85]
Randomized, controlled, parallel, double-blind (8 wk)	T1: plum juice with high dose anthocyanins (201 mg) T2: plum juice with low dose anthocyanins (47 mg) C: apricot juice	250 mL/d	Mild Cognitive Impairment of amnesic type	31 (19)	(n.d.)	T1 vs. C: TNF-α ↓ T1 vs. T2: TNF-α ↓ T1 vs. T2 vs. C: IL-6, IL-1β ↔	[86]
Randomized, controlled, parallel, double-blind (4 months)	T: cranberry juice C: placebo	230 mL, twice/d	Peripheral endothelial dysfunction and cardiovascular risk factors	69 (n.d.)	(n.d.)	T vs. C: TNF-α, IL-6 ↔	[69]
Randomized, controlled, crossover, blinded (5 d × 2)	T: Jerte Valley cherry-based product C: soft drink	125 mL twice/d	Young (20–30 y.o.) vs. middle-aged (35–55 y.o.) vs. elderly (65–85 y.o.); normal weight/overweight; healthy	30 (15)	All age groups: T: IL-1β, TNF-α and IL-8 ↑ C: IL-1β, TNF-α and IL-8 ↔	(n.d.)	[80]
Randomized, controlled, parallel (20 d)	T: tomato juice C: water	330 mL/d	Women; overweight/obese	104 (104)	T: TNF-α, IL-8 ↓; IL-6 ↔ C: TNF-α, IL-8 ↑; IL-6 ↔ T (overweight): TNF-α, IL-8 ↓; IL-6 ↔ T (obese): IL-6, IL-8, TNF-α ↔	T vs. C: TNF-α, IL-8 ↓; IL-6 ↔ T (overweight) vs. C: TNF-α, IL-8 ↓; IL-6 ↔ vs. T (obese) vs. C: IL-6, IL-8, TNF-α ↔	[83]
Randomized, controlled, crossover, double-blind (4 wk × 2)	T: bayberry juice C: placebo	250 mL twice/d	Young adults (18–25 y.o.); overweight/obese; 2/3 diagnostic criteria of fatty liver disease	44 (32)	T: TNF-α, IL-8 ↓ C: TNF-α ↑; IL-8 ↔	T vs. C: IL-8, TNF-α ↓	[75]
Randomized, controlled, parallel (7 d)	T: camu-camu juice, corresponding to 1050 mg of vitamin C C: 1050 mg of vitamin C	70 mL/d (T)	Male, habitual smokers, healthy	20 (0)	T: IL-6, IL-8 ↓ C: IL-6, IL-8 ↔	(n.d.)	[76]
Randomized, controlled, parallel (4 wk)	T: bilberry juice C: water	330 mL/d	High risk of cardiovascular disease	32 (n.d.)	(n.d.)	T vs. C: IL-6, IL-15, MIG ↓; TNF-α ↑; IL-1β, IL-1α, IL-1ra, IL-2, IL-2R, IL-4, IL-5, IL-7, IL-8, IL-10, IL-12, IL-13, IL-17, IFN-α, IFN-γ, GM-CSF, MIP-1α, MIP-1β, IP-10, MCP-1, Eotaxin, RANTES ↔	[72]
Randomized, controlled, parallel (12 wk)	T: cherry juice C: apple juice	200 mL/d	Normal weight/overweight; mild-to-moderate Alzheimer’s type dementia	42 (n.d.)	(n.d.)	T vs. C: IL-6 ↔	[79]
Randomized, controlled, crossover, single blind (8 wk × 2)	T: chokeberry (juice and oven-dried powder) C: placebo	300 mL/d juice and 3 g/d powder	Adults; mild hypertension	37 (n.d.)	(n.d.)	T vs. C: IL-10, TNF-α ↓ IL-4, IL-5, IL-6, IL-7, IL-8, IL-13, GM-CSF ↔;	[73]
Randomized, controlled crossover (4 wk × 2)	T: tart cherry juice C: anthocyanin-free fruit punch	240 mL/d	Overweight/obese	10 (8)	(n.d.)	T vs. C: TNF-α, IL-6, IL-10 ↔; MCP-1 ↓	[78]
Randomized, controlled, parallel, unblinded (5 d)	T: pomegranate juice C: water	220 mL/d	Unstable angina or myocardial infarction	T: 25 unstable angina + 25 myocardial infarctions C: 25 unstable anginas+ 25 myocardial infarctions TOT 100 (46)	(n.d.)	T vs. C: IL-6, TNF-α ↔	[87]
Randomized, crossover (4 wk × 2)	T1: vitamin C-rich apple juice T2: polyphenol-rich apple juice	250 mL, twice/d	Young adults (21–29 y.o.); normal weight/overweight; healthy	20 (12)	T1: IL-6, IL-8, IL-10, MCP-1 ↔ T2: IL-6, IL-8, IL-10, MCP-1 ↔	T1 vs. T2: IL-6, IL-8, IL-10, MCP-1 ↔	[82]
Comparative (8 wk)	T: tomato juice C: usual diet	1 serving/d, 4 times/wk	Metabolic syndrome	27 (3)	T: IL-6 ↔, TNF-α ↓ C: IL-6, TNF-α ↔	(n.d.)	[84]
Randomized, controlled, parallel (8 wk)	T: cranberry beverage C: water	750 mL/d	Gingivitis	45 (38)	T: IL-1β ↔ C: IL-1β ↔	(n.d.)	[71]

Legend: ↓, significant decrease; ↑, significant increase; ↔, no significant changes; C, control; d, days; GM-CSF, granulocyte-macrophage colony-stimulating factor; IFN-α, interferon alpha; IFN-γ, interferon gamma; IL, interleukin; IL-1α, interleukin 1 alpha; IL-1β, interleukin 1 beta; IL-1ra, interleukin 1 receptor antagonist; IL-2, interleukin 2; IL-2R, interleukin 2 receptor; IL-4, interleukin 4; IL-5, interleukin 5; IL-6, interleukin 6; IL-7, interleukin 7; IL-8, interleukin 8; IL-10, interleukin 10; IL-12, interleukin 12; IL-13, interleukin 13; IL-15, interleukin 15; IL-17, interleukin 17; IL-18, interleukin 18; IFN-γ, interferon gamma; IP-10, interferon gamma-induced protein 10; MCP-1, monocyte chemoattractant protein 1; MIG, monokine-induced by gamma interferon; MIP-1α, macrophage inflammatory protein 1 alpha; MIP-1β, macrophage inflammatory protein 1 beta; n.d., not declared; RANTES/CCL5, regulated upon activation normal T cell expressed and secreted/chemokine ligand 5; T, treatment; TNF-α, tumor necrosis factor alpha; wk, weeks.

Bilberries were evaluated in participants at high risk for cardiovascular disease over a four-week period. The results showed significantly lower IL-6, IL-15, and MIG levels, but higher TNF-α levels, in the treatment group compared to the control group (water). the other 22 markers were not significantly different between the groups [72]. Mildly hypertensive adults aged 40–70 years were treated with either chokeberry or a placebo for 8 weeks. Results showed no significant differences between groups for IL-4, IL-5, IL-6, IL-7, IL-8, IL-13, or GM-CSF. However, the treatment group exhibited significantly lower IL-10 and TNF-α levels [73]. In a study by Amagase and coworkers [74], older adults were administered with 60 mL goji fruit juice twice a day for 30 days. The levels of IL-2 in the treatment group significantly increased vs. baseline and were higher compared to the placebo group, while IL-4 levels remained unchanged. In a 4-week study by Guo and collaborators [75], young overweight or obese adults with one or more diagnostic criteria for fatty liver disease were administered with bayberry juice. The treatment group showed significant reductions in both TNF-α and IL-8 levels compared to baseline, while the placebo group experienced an increase in TNF-α and no significant change in IL-8. Moreover, the bayberry group showed significantly lower TNF-α and IL-8 levels compared to the control group. Then, it was observed that 70 mL of camu-camu juice daily for 7 days, compared to an equivalent dose of vitamin C (1050 mg), reduced IL-6 and IL-8 levels in male, habitual smokers [76].

The three studies evaluating cranberry juices did not observe any change in the TNF-α or IL-6 levels of participants with peripheral endothelial dysfunction and cardiovascular risk factors [69]. Moreover, IL-6 levels were not changed by low-energy cranberry juice (daily, 8 weeks) in women with metabolic syndrome [70]. A lack of effect on IL-1β was observed also for patients with gingivitis [71].

As regards cherries, three out of four studies detected a shift in cytokine levels. The study by Arbizu and coworkers [77] assessed the impact of 200 mL of dark sweet cherry juice supplemented with cherry powder twice daily in obese participants over 30 days. The study found no significant changes in most inflammatory markers between baseline and post-intervention within either cherry or placebo groups. Conversely in the placebo group, IL-1ra decreased and IL-10 increased with respect to baseline. When comparing the treatment and control groups, the results showed that the treatment group had significantly lower levels of IL-10 and IFN-γ, while IL-1β was lower in the treatment group compared to controls. In the study conducted by Martin and colleagues [78], the effect of tart cherry juice (240 mL/day for 4 weeks) was compared to an anthocyanin-free fruit punch (control). The results indicated no significant differences in TNF-α, IL-6, and IL-10 between the groups, except for lower MCP-1 levels in the treated group. A cherry-based product was compared to a soft drink in a healthy, non-obese population that included young, middle-aged, and elderly participants. The intervention lasted for 5 days, with participants consuming 125 mL of the drinks twice daily. Results showed that the cherry-based product induced an increase in levels of IL-1β, TNF-α, and IL-8 across all age groups [80].

When cherry juice was compared to apple juice as a control (200 mL/day, 12 weeks) in participants with mild-to-moderate Alzheimer’s type dementia, no significant differences in IL-6 levels between groups were observed [79].

Two studies evaluating the effect of apple juice did not observe significant changes in cytokines within or between both the treatment and control groups. Specifically, overweight/obese men did not experience changes in TNF-α or IL-6 levels [81], and also there were no changes in IL-6, IL-8, IL-10, and MCP-1 levels in healthy, non-obese young adults [82]. On the contrary, both studies on tomato juice detected a modulation of cytokine levels. When overweight and obese women were given 330 mL/day of tomato juice or water for 20 days, their TNF-α and IL-8 levels significantly decreased following juice consumption, while their IL-6 levels remained unchanged. Conversely, in the control group, TNF-α and IL-8 levels increased, while IL-6 levels remained unchanged. When comparing groups, the tomato juice group had significantly lower TNF-α and IL-8 levels compared to the control, particularly in overweight women, but not obese women [83]. Similar results were observed when the effects of one serving/day of tomato juice were assessed in individuals with metabolic syndrome over a period of 8 weeks. The treatment induced a significant reduction in TNF-α levels, while IL-6 levels remained unchanged [84]. However, no data were available regarding the serving sizes.

Modulations in cytokines were observed also following treatments with plums, red oranges, and pomegranates. The study conducted by do Rosario and colleagues [86] investigated the effects of naturally anthocyanin-rich plum juice on individuals with mild cognitive impairments of amnesic type. Participants were divided into three groups: one receiving plum juice with a high dose of anthocyanins (201 mg), another with a lower dose (47 mg), and a control group receiving apricot juice. Results showed that TNF-α levels, but not IL-6 and IL-1β, were significantly lower in the high-dose anthocyanin group compared to both the low-dose and control groups. Participants with increased cardiovascular risk consumed 250 mL of red orange juice twice a day for one week. The results indicated a reduction in TNF-α and IL-6 levels in the treatment group [85]. When participants with unstable angina or myocardial infarction were administered 220 mL of pomegranate juice or water daily for 5 days, results showed no significant differences between the groups in terms of IL-6 and TNF-α levels [87].

#### 3.2.2. Hot Beverages

The seven studies summarized in Table 9 involved hot beverages: moka coffee [88] and a comparison between caffeinated and decaffeinated coffee [89], tea [90,91], herbal infusions [92], chicory coffee [93], and cocoa/milk [94]. The total number of participants involved in these studies was 221. Four out of seven studies reported reduced cytokine levels, one reported increased levels, and two showed no variations.

**Table 9:** **Summary of randomized controlled trials investigating the effects of hot beverages**. Each study includes details of the design, duration, treatment, dose, study population, and reported outcomes. Results are presented as changes within groups and between groups.

Study Design (Duration)	Treatment	Dose	Study Population	Study Population Number (F)	Results Within Groups	Results Between Groups	References
Randomized; controlled; parallel, single blind (8 wk)	T1: green tea T2: green tea extract capsules in water C: water	Tea or water: 4 cups/d Extract: 2 capsules	Obese; metabolic syndrome	29 15 trios in two years.	(n.d.)	T1 vs. C: adiponectin, IL-6, IL-1β ↔ T2 vs. C: adiponectin, IL-6, IL-1β ↔	[91]
Randomized, controlled, parallel, double blind (4 wk)	T: fruit concentrates in the herbal beverages of chamomile, meadowsweet, and willow bark C: fruit concentrates in water	250 mL/d	Healthy	20 (n.d.)	(n.d.)	T vs. C: TNF-α, IL-1β, IL-6 ↔	[92]
Single arm, 3-stages, single-blind (4 wk × 3)	Moka coffee	C: no coffee T1: 150 mL × 4/d T2: 150 mL × 8/d	Elevated risk of type 2 diabetes	47 (36)	(n.d.)	T1 vs. C: IL-6, MIF, IL-1ra, IL-18, adiponectin ↔ T2 vs. C: IL-6, MIF, IL-1ra ↔; IL-18 ↓; adiponectin ↑	[88]
Self-controlled (8 d)	T: soluble mate tea C: water	200 mL × 3/d	Men (25.0 ± 3.0 y.o.), healthy	9 (0)	(n.d.)	T vs. C: IL-1β ↔; TNF-α, IL-6 ↓	[90]
Randomized, controlled, parallel (8 wk)	T1: caffeinated coffee T2: decaffeinated coffee C: water	T1, T2: 2 g in 177 mL water, 5 times/d C: 177 mL water, 5 times/d	Overweight/obese; healthy	45 (29)	(n.d.)	T1 vs. C: IL-6 ↑ T2 vs. C: IL-6 ↑ T1 vs. T2: IL-6 ↔	[89]
Randomized, controlled, cross-over (4 wk × 2)	T: fiber-rich cocoa product in semi-skimmed milk C: semi-skimmed milk	T: 15 g in 200 mL twice/d C: 200 mL twice/d	Healthy vs. moderately hypercholesterolemic (non-obese)	44 (24)	T: TNF-α, IL-1β, IL-6, IL-8, MCP-1 ↔; IL-10 ↓ C: TNF-α, IL-1β, IL-6, IL-8, MCP-1, IL-10 ↔	(n.d.)	[94]
Self-controlled (1 wk)	Chicory coffee	300 mL/d	Healthy	27 (13)	MIF ↓	(n.d.)	[93]

Legend: ↓, significant decrease; ↑, significant increase; ↔, no significant changes; C, control; d, days; n.d., not declared; IL, interleukin; IL-1β, interleukin 1 beta; IL-6, interleukin 6; IL-10, interleukin 10; IL-18, interleukin 18; MCP-1, monocyte chemoattractant protein 1; MIF, macrophage migration inhibitory factor; n.d., not declared; T, treatment; TNF-α, tumor necrosis factor alpha; wk, weeks.

Studies involving coffee showed a modulation in cytokine production. Individuals at elevated risk of T2D followed three four-week steps: they first avoided coffee (control; T0), then consumed 150 mL of moka coffee four times daily (T1), and, lastly, they consumed the same quantity eight times daily (T2). At T2, participants showed lower circulating levels of IL-18 and higher levels of adiponectin and IL-6 with respect to the control group (T0); macrophage migration inhibitory factor (MIF) and IL-1ra did not change. During the T1 stage, cytokines were not significantly modified [88]. In an 8-week study by Wedick and coworkers [89], overweight and obese individuals were divided into three groups: one consuming soluble caffeinated coffee (2 g in 177 mL of water, 5 times/day), one consuming the same amount of decaffeinated coffee, and a control group consuming the same quantity of water (control). The results showed a significant increase in IL-6 levels in both the caffeinated and decaffeinated coffee groups compared to the control, without showing significant differences between the two coffee groups.

Three studies investigated the effects of infusions. In a two-step study by Panza and coworkers [90], healthy young men consumed at first 200 mL of water (control) three times daily for 8 days, then the same quantity of mate tea for another 8 days. After the tea intervention, lower TNF-α and IL-6 levels were observed, while no significant differences were found for IL-1β levels. In another 8-week study, obese individuals with metabolic syndrome were divided into three groups: one consuming four cups of green tea, another consuming two capsules of green tea extract in water, and a control group drinking water. No significant differences were observed between the treatment groups and the control group in terms of adiponectin, IL-6, or IL-1β levels [91]. In a 4-week study by Drummond and coworkers [92], healthy participants consumed a daily serving of 250 mL of either a fruit concentrate beverage combined with herbal ingredients such as chamomile, meadowsweet, and willow bark (treatment group) or fruit concentrates in water (control group). The results showed no significant differences between the treatment and control groups in TNF-α, IL-1β, and IL-6 levels.

Two studies evaluated the effect of chicory coffee [93] and cocoa [94], reporting, respectively, a decrease in macrophage MIF compared to baseline after one week in healthy individuals and a decrease only in IL-10 in non-obese participants who were either healthy or moderately hyper-cholesterolemic.

### 3.3. Summary of Cytokine Modulation Across Food Groups and Typology of Subjects

Table 10 summarizes the direction of cytokine variations observed in response to different food categories, considering all studies together and stratified by the health status of participants. For this purpose, studies were classified as “healthy” (HS) when participants were explicitly described as healthy or when no risk factors were reported and as “non-healthy” when participants presented any type of pathology or risk factor, including older age, smoking, obesity, or other conditions (SPRF). Among pathologies, the most common conditions were obesity, metabolic syndrome, and cardiovascular diseases. Cytokine responses were categorized as follows: “no changes” when no cytokine showed a significant change, either versus baseline or between groups; “higher” when at least one cytokine increased significantly in any intervention group, regardless of whether the change was versus baseline or versus a control group; and “lower” when at least one cytokine decreased significantly under the same criteria. In cases where the same intervention induced opposite cytokine responses (i.e., both increases and decreases depending on the cytokine measured), the result was split into separate study entries. The number of studies with healthy subjects (*n* = 8 vs. *n* = 5, respectively, for solid foods and beverages) are lower compared to the studies with subjects with pathologies (*n* = 44 vs. *n* = 23, respectively, for solid foods and beverages).

Overall, solid foods were ineffective in modifying cytokine levels in 20 out of the 57 studies (35%; 20/57), with nuts in first place (71%; 5/7), followed by oils and fats (43%; 3/7), cereals (36%; 4/11), dairy (33%; 2/6), miscellaneous (29%; 2/7), and, lastly, fruits and vegetables (20%; 2/10). However, cereals and fruits and vegetables showed an efficiency in reducing circulating cytokines in healthy subjects, respectively, of 67% (2/3) and 50% (1/2), while fish, dairy, and miscellaneous showed a 100% reduction in the only study conducted with each food type. No study displayed an increase in cytokine levels in healthy people.

When the studies were conducted on subjects with pathologies or risk factors, fruits and vegetables displayed the highest efficiency in reducing cytokines levels (75%; 6/8), followed by oils and fats (57%; 4/7) and cereals and fish (50%; 4/8), while dairy, miscellaneous, and seeds and nuts were efficient only in 40%, 33%, and 29% of studies, or in 2/5, 2/6, and 2/7 studies, respectively. It is noteworthy that, except for in oils and fats and seeds and nuts, increases in cytokine levels were also observed. This occurred in 1/11 cases (9%) for cereals, 1/10 (10%) for fruits and vegetables, 2/9 (22%) for fish, 1/6 (17%) for dairy, and 2/7 (29%) for miscellaneous foods. However, most of the cytokines that increased in cereals, fruits and vegetables, and fish were anti-inflammatory, namely IL-10 (cereals and in fish) and IL-1ra (fruits and vegetables). IL-10 also increased after consumption of vegetable soup (miscellaneous foods). Dairy products, in turn, led to an increase in both the anti-inflammatory cytokine adiponectin and the pro-inflammatory cytokines IFN-γ and IL-12. Only one food, honey, induced an increase in the pro-inflammatory cytokine TNF-α.

Beverages were ineffective in modifying cytokine levels in 32% of cases (9/28). Specifically, fruit and vegetable juices were effective in 35% (7/20) of cases and hot beverages in 25% (2/8). Fruit and vegetable juices reduced cytokine levels only in SPRF (56%; 10/18), while hot beverages were effective in HS (67%; 2/3) and SPRF (50%; 2/5). No anti-inflammatory effects were displayed when fruit and vegetable juices were provided to HS.

However, an increase in cytokine levels was described in 3/20 cases (15%) for fruit and beverage juices and in 2/8 cases (25%) for hot beverages with a total of 18% (5/28) for both juices and beverages. Differently from what we observed for solid foods, the increases in cytokines following dietary treatment were mainly pro-inflammatory, including IL-1β, IL-2, IL-6, IL-8, and TNF-α, with the only exception being the anti-inflammatory marker adiponectin, which increased after coffee consumption in SPRF.

### 3.4. Risk of Bias

**Figure 2:**
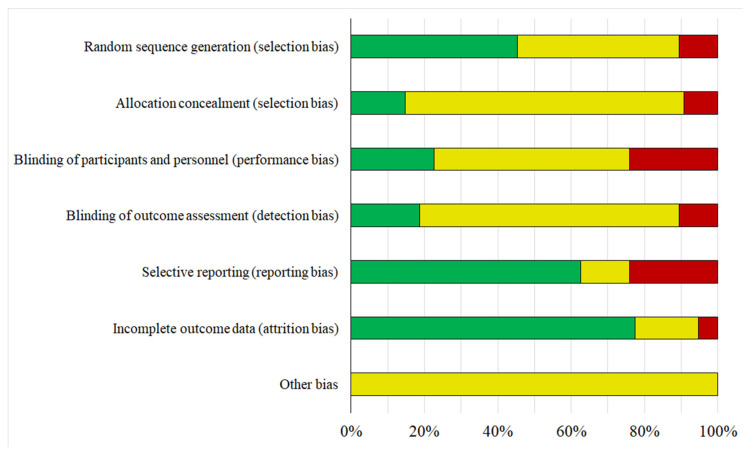
Risk of bias graph presented as percentages across all included studies. •: low risk, •: unclear, •: some or high risk of bias.

**Figure 3:**
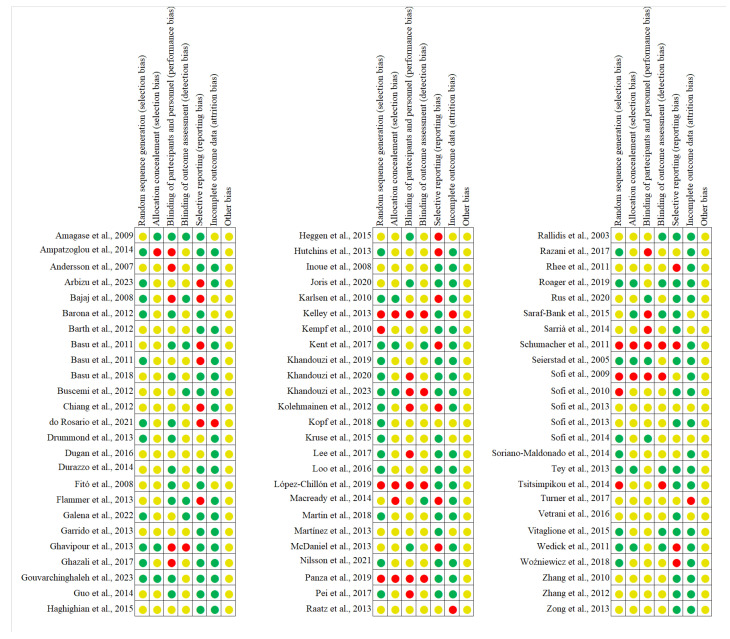
Risk of bias graph for all the included studies. •: low risk, •: unclear, •: some or high risk of bias.

The evaluation of the risk of bias, shown in Figure 2 and Figure 3, identified further strengths and weaknesses in the considered studies. Many studies demonstrated appropriate data handling, with low risks of selective reporting (reporting bias) and incomplete outcome data (attrition bias). Randomization was performed in the majority of the studies, but information on sequence generation was often omitted, increasing the possibility of selection bias. Allocation concealment (selection bias) and detection bias (blinding of outcome assessment) were generally rated as moderate to high risk, primarily due to insufficient reporting. Similarly, performance bias (blinding of participants and personnel) was a common concern, mainly because the nature of the interventions made it difficult to fully conceal treatment assignments.

## 4. Discussion

The present work shows, for the first time, the results of a systematic review of the literature concerning the role of foods, meaning solid foods and beverages, on markers of systemic inflammation, both in HS and SPRF. Data showed that there was a significant reduction in circulating cytokine levels and/or an increase in anti-inflammatory cytokines (80% of observed instances; 8/10) in subjects administered with fruits and vegetables, followed by fish (78%; 7/9), dairy (67%; 4/6), cereals (64%; 7/11), and oils (57%; 4/7). Concerning fruit and vegetable beverages as well as hot beverages, they both showed a decrease in circulating cytokines in 50% of cases (10/20 and 4/8, respectively). However, when an increase in cytokine levels was observed for solid foods, the involved cytokines were mostly the anti-inflammatory ones, except for in one study where honey—20 g daily for 12 weeks—was given [66]. On the contrary, increases in circulating cytokines following beverage consumption were driven mostly by pro-inflammatory cytokines. As further findings, we also observed higher efficiencies when studies were conducted in SPRF supplemented with fruits and vegetables (87.5%; 7/8), fish (75%; 6/8), and cereals (62.5% 5/8). This latest finding is in agreement with our previous systematic review on the role of flavonoid-rich foods in modulating inflammatory markers in human studies [95] where we showed that none of the intervention studies (0/21) conducted in healthy subjects were effective in reducing levels of TNF-α after ingestion of flavonoid-rich foods or relative supplements. On the other hand, in cases of subjects characterized by risk factors for CVD, flavonoids decreased TNF-α in almost 30% of the interventions (5/17). The effect was even more pronounced when the studies were conducted on subjects with pathologies, with 60% of the interventions (4/7) showing a decrease in levels of TNF-α after supplementation either with papaya, soy products, green tea extract or 1-year adherence to the Mediterranean diet [95].

Moreover, also in meta-analyses by Lettieri Barbato et al. [96], results from dietary intervention studies using plant foods to modulate antioxidant status in humans showed that the antioxidant effects of most foods and beverages were more effective in subjects with pathologies, with an effect size of 0.937 (*p* < 0.001) with respect to the results in healthy subjects (effect size 0.367; *p* < 0.05). This was also reported in the PREDIMED study, a clinical intervention trial that compared a diet of olive oil and a mixture of nuts to a low-fat diet in subjects with at least three risk factors for CVD [97]. Supplementation for one year with olive oil or nuts induced a higher increase in plasma levels of antioxidants in subjects in the first quartile (lower plasma levels), with respect to subjects starting from higher baseline levels (4th quartile), of plasma antioxidant capacity; the latter of whom did not experience any statistically significant increases [97]. Further indirect evidence came from the EPIC study, where the protective effect of dietary antioxidant intake against gastric cancer risk, characterized by ongoing oxidative and inflammatory stress, was more effective in smokers than in non-smokers [98]. Taken together, this evidence highlights the paramount importance of an increased intake of plant foods in conditions where inflammatory and oxidative stress is present, while in healthy subjects the bioactive ingredients present in the food matrix have the role of maintaining a homeostatic balance associated with wellbeing and disease prevention [98].

In order to understand the pro-(anti)-inflammatory role of foods, it is important to recall the main mechanism involved in the rise in inflammatory conditions. The consumption of high-energy or nutritionally unbalanced meals causes postprandial stress in the body, characterized by intense metabolic and immune activity aimed at efficiently metabolizing lipids, carbohydrates, proteins and other nutrients, and neutralizing potentially toxic or unwanted compounds [99]. Frequent ingestion of HFM or available carbohydrates (High Carbohydrate Meals, HCM), in addition to increasing circulating levels of classic risk factors, such as lipids and glucose, triggers a systemic inflammatory/oxidative response that induces an increase in cardiovascular risk factors and, if prolonged over time, causes a greater predisposition to degenerative diseases [100]. In particular, during the postprandial phase, an increase in the number of leukocytes, especially granulocytes, and in the expression levels of neutrophil and monocyte activation markers were observed, with consequent production of pro-inflammatory cytokines and free radicals [101]. This condition begins as early as 30 min after meal consumption, suggesting that the entire body is actively engaged during the postprandial period, when the body is in a permanent state of “metabolic stress” up to approximately 6–8 h after eating a meal, depending on the calories and macro-nutrients ingested [15]. If postprandial stress events are occasional and limited in duration, the body manages the inflammatory response, returning to a state of equilibrium. If, on the other hand, stressful meals represent a continuous event considering the number of meals consumed daily, the 6–8 h postprandial stress condition can extend for most of the day and, consequently, become chronic over time [15]. Furthermore, metabolic responses to the same meal take on very different meanings depending on an individual’s characteristics. In subjects with metabolic syndrome, diabetes, or obesity, characterized by a chronic baseline inflammatory status, inflammatory responses to meals are much more pronounced and prolonged, and therefore potentially more harmful, than in subjects without pre-existing inflammatory conditions, such as those with normo-weight [102].

It is therefore crucial to prevent the onset of chronic metabolic stress by avoiding the prolonged consumption of repeatedly high-calorie and nutritionally unbalanced meals or by adopting measures that reduce stress at each meal. In this regard, available evidence suggests the importance of pairing a “stress-inducing” meal with plant-based foods or drinks. Plant foods, thanks to their bioactive molecules, help the body hamper inflammatory and oxidative responses and the associated damage [16,103]. The presence of antioxidant and anti-inflammatory compounds, such as polyphenols, in plant-based foods may underlie this protective effect [104]. Their presence in combination with a stressful meal ensures an effective antioxidant and anti-inflammatory effect, regardless of their absorption in biological fluids, thanks to a “sacrificial” effect during the digestive process in the stomach bioreactor due to their high concentration in foods (millimolar). This effect would not be as effective if foods are eaten not in association with the meals due to the low concentration of polyphenols in biological fluids (nano to micromolar) and their metabolic transformation and high excretion rates from the body [105,106].

All the studies reviewed here did not give advice on how and when to eat specific foods, i.e., during or long before/after meal consumption, probably reducing the anti-inflammatory efficiency of the bioactive ingredients. Future studies should consider the main driving forces leading to food-related inflammatory conditions, such as postprandial stress, suggest the consumption of tested foods during the meal and not outside of meals. This procedure will probably allow for the amplification of the anti-inflammatory effect and take full advantage of the wide array of nutritional and non-nutritional (i.e., flavonoids) bioactive plant food components within healthy diets.

The most extensively investigated food groups were fruits and vegetables, cereals (mainly whole grains), oils, seeds, and fish. The emphasis on fruits, vegetables, cereals, and mono-/poly-unsaturated-based dietary fats aligns with existing dietary guidelines that advocate for a diet rich in plant-based foods to support immune and metabolic health [9,107]. Despite findings from in vitro and animal studies suggesting that bioactive compounds in fruits, vegetables, and legumes exhibit anti-inflammatory properties [108], human data still remains limited. Interestingly, despite the widespread recognition of fruits and vegetables as anti-inflammatory foods, few clinical studies have focused on them; however, results from this work are extremely promising, indicating a high-efficiency anti-inflammatory effect mediated by a decrease in pro-inflammatory cytokines and an increase in anti-inflammatory ones. Focusing on the type of fruits and vegetables provided to subjects, due to the low number of studies, it is hard to identify single foods responsible for this anti-inflammatory effect; however, it is important to note that berries, specifically freeze-dried grape powder [32], freeze-dried strawberries [33], cherry [34] puree, and dried bilberries [35], showed anti-inflammatory effects in all the studies.

A relatively high number of studies focused on fruit-based drinks. However, even though fruits and vegetables displayed a high efficiency in reducing systemic inflammation—which was more pronounced in subjects with pathologies—beverages were more likely to increase pro-inflammatory cytokine levels than their solid counterparts. This negative effect might be explained by the high sugar and/or additive content present in fruit juices; furthermore, the nutrient content of the juices provided is lacking [109]. In agreement with this hypothesis, it is worth noting that the only study with solid foods that increased levels of pro-inflammatory cytokines was conducted with a quite high and unrealistic amount of honey, i.e., 20 g for 12 weeks, in chronic smokers [66]. Despite the fact that there is not yet conclusive evidence of this, it seems that when simple sugars are present, pro-inflammatory cytokine production takes place.

Overall, solid foods have been investigated in a higher number of studies with respect to beverages (53 vs. 26 interventions, respectively) mainly due to their presence in traditional dietary patterns, such as the Mediterranean diet, associated with disease prevention. In the instance of fruits, which can be consumed both as foods or as juices, this emphasis on beverages can be easily explained by their practicality as consistent and easily standardized interventions, making them particularly appealing for clinical trials.

Notably, olive oil, flaxseed, and salmon are among the most frequently cited foods. Their prevalence is probably due to their richness in mono-/poly-unsaturated fatty acids, which are generally associated with various health benefits [110,111]. Among the seven fish-based interventions, cytokine reductions were observed in approximately 71.4% of studies (5/7), particularly following oily fish consumption, while oils showed effects in 57% of the studies (4/7). In contrast, flaxseeds, the most extensively investigated food within this category, reported no significant effects.

As regards cereal-based products, approximately 55% of cases (6/11) showed a decrease in cytokine levels, with around 36% reporting no significant changes. The lack of a uniform definition of “whole grain” leads to variability in whole-grain product composition, such as grain type, processing methods, and the proportion of ingredients [112], which may explain the variability of the outcomes. Two studies focusing on individuals with a previous low intake of whole grains [25,26] reported a reduction in cytokine levels following whole grain consumption, suggesting that the observed effect may be influenced by a lack of adherence to dietary recommendations.

Overall, the studies included in this review varied widely in design, doses, randomization, and use of appropriate control groups, making it challenging to draw consistent conclusions. First, interventions were conducted over time frames ranging from just a few days to several months, which complicated our ability to assess how quickly or consistently dietary components might influence inflammatory markers. Another challenge came from differences in the control conditions used across studies. Some studies compared the effects of foods to participants’ usual diets, while others used carefully designed control foods that were nearly identical to the test foods, differing only in the specific component being studied. This lack of standardization makes it harder to compare results and interpret them meaningfully. It is also noteworthy that some studied did not include control groups. Well-controlled and carefully designed studies are essential for isolating the specific effects of dietary components on inflammation [113]. Without appropriate control conditions and standardized methodologies, assessing whether observed changes in cytokine levels are truly attributable to dietary interventions or influenced by external factors is not feasible.

Beyond the challenges linked to the study design, the choice of cytokines assessed through the studies is worth considering. Although a total of 31 different cytokines were analyzed across the studies, certain cytokines were investigated far more frequently than others. IL-6 was the most studied, appearing in 61 studies, followed by TNF-α in 47 studies. This highlights a significant gap, as the third most cited cytokine, IL-10, was examined in only 17 studies—far fewer than the top two. The frequent investigation of IL-6 and TNF-α reflects general interest regarding their role in immune function and disease processes. These cytokines have been commonly used as biomarkers to assess the inflammatory effects of diets [114], foods [115], and supplements [116,117]. However, several potentially important inflammatory markers remain largely unexplored. Future studies should adopt a more comprehensive approach by analyzing broader cytokine patterns to construct a more detailed overview of the inflammatory landscape.

Overall, unclear or missing methodological details were observed across multiple domains, contributing to a moderate to high risk of bias in most of the areas (Figure 2 and Figure 3). This highlights the need for caution when interpreting the overall findings of the selected studies. Standardization of study design, including the use of well-defined control conditions and longer intervention trials, is crucial for improving the reliability and robustness of findings.

A potential limitation of this study must be acknowledged: our selection criteria led to the exclusion of studies involving fortified foods or dietary interventions associated with physical activity. However, the reasons for our choice rely on needing to investigate the effect of single food items without any further intervention. Information on food origin, production methods, level of processing, and the nutritional and bioactive content of foods was not consistently reported across studies, preventing a discussion on the roles of these variables on the anti-inflammatory role of foods.

## 5. Conclusions

The current evidence suggests that specific food groups, such as fruits and vegetables (specifically berries), fish, and cereals, may reduce systemic inflammation, mainly in subjects with pathologies or risk factors. However, inconsistencies in study design and methodology, along with a limited number of studies, prevent us from drawing solid conclusions. This review emphasizes the need for well-designed, standardized studies to strengthen current evidence on the complex relationship between foods and beverages and inflammatory markers. Dietary intervention trials encompassing broader cytokine patterns, including pro- and anti-inflammatory cytokines, resembling the real in vivo situation of the overall inflammatory response are urgently needed to understand the role of foods in modulating low-grade chronic inflammation and to deliver findings to the general public. In this view, despite the massive amount of information from social media, newspapers, and experts highlighting the role of specific foods in modulating inflammatory responses, evidence from human trials is limited and recommendations should be taken with caution to avoid delivering inaccurate advice to consumers, including our results showing potential pro-inflammatory effects in some studies.

## Figures and Tables

**Table 10:** **Changes in cytokine circulating levels in the dietary intervention studies are expressed as the percentage of studies for each food category. Results are divided into all subjects, healthy subjects, and subjects with pathologies or risk factors**. Data are grouped for food or beverage category and classified based on whether cytokines did not change at all; increased (at least one), or decreased (at least one) significantly (*p* < 0.05; *p* < 0.01; and *p* < 0.001) following dietary interventions. When, in the same study, cytokines increase and decrease, both results are reported. The percentage of studies reporting each outcome within each category is indicated.

	All			Healthy Subjects (HS) *			Subjects with Pathologies or Risk Factors (SPRF) *		
	No Changes	Higher Cytokine Levels	Lower Cytokine Levels	No Changes	Higher Cytokine Levels	Lower Cytokine Levels	No Changes	Higher Cytokine Levels	Lower Cytokine Levels
Cereals	4/11 (36%)	1/11 (9%)	6/11 (55%)	1/3 (33%)	(n.p)	2/3 (67%)	3/8 (38%)	1/8 (13%)	4/8 (50%)
Fruits and Vegetables	2/10 (20%)	1/10 (10%)	7/10 (70%)	1/2 (50%)	(n.p)	1/2 (50%)	1/8 (13%)	1/8 (13%)	6/8 (75%)
Oils and Fats	3/7 (43%)	(n.p)	4/7 (57%)	(n.p)	(n.p)	(n.p)	3/7 (43%)	(n.p)	4/7 (57%)
Seeds and Nuts	5/7 (71%)	(n.p)	2/7 (29%)	(n.p)	(n.p)	(n.p)	5/7 (71%)	(n.p)	2/7 (29%)
Fish	2/9 (22%)	2/9 (22%)	5/9 (56%)	(n.p)	(n.p)	1/1 (100%)	2/8 (25%)	2/8 (25%)	4/8 (50%)
Dairy	2/6 (33%)	1/6 (17%)	3/6 (50%)	(n.p)	(n.p)	1/1 (100%)	2/5 (40%)	1/5 (20%)	2/5 (40%)
Miscellaneous	2/7 (29%)	2/7 (29%)	3/7 (43%)	(n.p)	(n.p)	1/1 (100%)	2/6 (33%)	2/6 (33%)	2/6 (33%)
Foods (total)	20/57 (35%)	7/57 (12%)	30/57 (53%)	2/8 (25%)	0/8 (0%)	6/8 (75%)	18/49 (37%)	7/49 (14%)	24/49 (49%)
Frut and Vegetable Juices	7/20 (35%)	3/20 (15%)	10/20 (50%)	1/2 (50%)	1/2 (50%)	(n.p)	6/18 (33%)	2/18 (11%)	10/18 (56%)
Hot beverages	2/8 (25%)	2/8 (25%)	4/8 (50%)	1/3 (33%)	(n.p)	2/3 (67%)	1/5 (25%)	2/5 (50%)	2/5 (50%)
Beverages (total)	9/28 (32%)	5/28 (18%)	14/28 (50%)	2/5 (40%)	1/5 (20%)	2/5 (40%)	7/23 (30%)	4/23 (17%)	12/23 (52%)
Total	29/85 (34%)	12/85 (14%)	44/85 (52%)	4/13 (31%)	1/13 (8%)	8/13 (62%)	25/72 (35%)	11/72 (15%)	36/72 (50%)

* Legend: HS, healthy subjects: participants explicitly described as healthy or when no risk factors were reported; n.p., not present; SPRF, subjects with pathologies or risk factors (SPRF): participants presented any type of pathology (obesity, metabolic syndrome and cardiovascular diseases, etc.) or risk factors (aging, smoking, dyslipidemia, etc.).

## Data Availability

The original contributions presented in this study are included in the article; further inquiries can be directed to the corresponding author.

## Abbreviations

The following abbreviations are used in this manuscript:

CVDs	Cardiovascular diseases
NCDs	Non-communicable diseases
T2D	Type 2 diabetes
TNFs	Tumor necrosis factors
ILs	Interleukins
IFNs	Interferons
TGF-β	Tumor growth factor β
HFM	High fat meal
MCP-1	Monocyte chemoattractant protein 1
GM-CSF	Granulocyte/monocyte-colony stimulating factor
MIF	Macrophage migration inhibitory factor
HS	Healthy subjects
SPRF	Subjects with pathologies or risk factors
